# Endoscopic Endonasal Approach for Transclival Resection of a Petroclival Meningioma: A Technical Note

**DOI:** 10.7759/cureus.641

**Published:** 2016-06-14

**Authors:** Walter C Jean, Daniel R Felbaum, Amjad Anaizi, Timothy R DeKlotz

**Affiliations:** 1 Neurosurgery, Medstar Georgetown University Hospital; 2 Otolaryngology, Medstar Georgetown University Hospital

**Keywords:** endoscopic, endonasal, petroclival, meningioma

## Abstract

The endoscopic endonasal transclival approach has been widely described for its use to resect clivus chordomas, but there have only been isolated reports of its use for petroclival meningiomas. These tumors are most often resected utilizing open transpetrosal approaches, but these operations, difficult even in the hands of dedicated skull base surgeons, are particularly challenging if the meningiomas are medially-situated and positioned mainly behind the clivus. For this subset of petroclival meningiomas, a transclival approach may be preferable. We report a meningioma resected via an endoscopic endonasal transclival technique. The patient was a 63-year-old man who presented originally for medical attention because of diplopia related to an abducens palsy on the left. A workup at that time revealed a meningioma contained entirely in the left cavernous sinus, and this was treated with stereotactic radiosurgery. His symptoms resolved and his meningioma was stable on MRI for several years after treatment. The patient was then lost to follow-up until 13 years after radiosurgery when he experienced intermittent diplopia again. At this point, workup revealed a large petroclival meningioma compressing the brainstem. He underwent a successful endoscopic endonasal transclival resection of this tumor. A demonstration of the step-by-step surgical technique, discussion of the nuances of the operation, and a comparison with the open transpetrosal approaches are included in our report.

## Introduction

The transpetrosal approaches have been long considered the most favored, if not “standard”, approach for resecting tumors of the petroclival region [1]. However, ever since it first appeared in the literature in 2005, the endoscopic endonasal transclival approach has significantly impacted the discourse on this topic [2]. There have been many recent reports about using this endoscopic technique for clival chordomas, and yet, only scattered reports exist regarding its application on petroclival meningiomas [3-4]. We aim to add to the body of evidence that supports the use of the endoscopic endonasal approach for these difficult meningiomas as well as to provide the first video demonstration of this technique.

## Technical report

Clinical presentation

**Figure 1:**
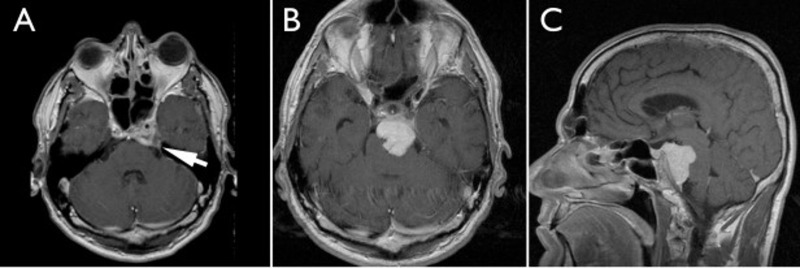
Preoperative Imaging A: Axial view, five years after radiosurgery for his left cavernous sinus meningioma. Arrow: area of the tumor. B: Axial view, 13 years after radiosurgery showing a large petroclival meningioma compressing the pons and engulfing the basilar artery. C: Coronal view, showing the same tumor in B, demonstrating the superior-inferior extent of the meningioma.

A 63-year-old man presented with several months of intermittent diplopia. He had similar symptoms 13 years ago and was diagnosed with a left abducens palsy related to a left cavernous sinus meningioma. He was treated with stereotactic radiosurgery at that time and his symptoms resolved. After several annual MRIs for observation, he was lost to follow-up. Because of the recurrence of his symptoms, he received a new MRI, which showed a large petroclival meningioma, extending from the left posterior clinoid to the level of the mid-clivus, with compression of the pons (Figure 1).

After obtaining informed consent, plans were made for an endoscopic endonasal transclival resection of his tumor. 

Operative technique

After induction of general anesthesia, the patient was positioned supine with the head in gentle extension and rotated slightly to the right. The stereotactic navigation camera was placed at the head of the bed and video towers on the patient’s left. During the approach, the sinus surgeon worked from the patient’s right; however, for the main portion of the procedure, when both surgeons worked together, the neurosurgeon worked from the patient’s right and the sinus surgeon controlled the camera from the head of the bed.

**Figure 2:**
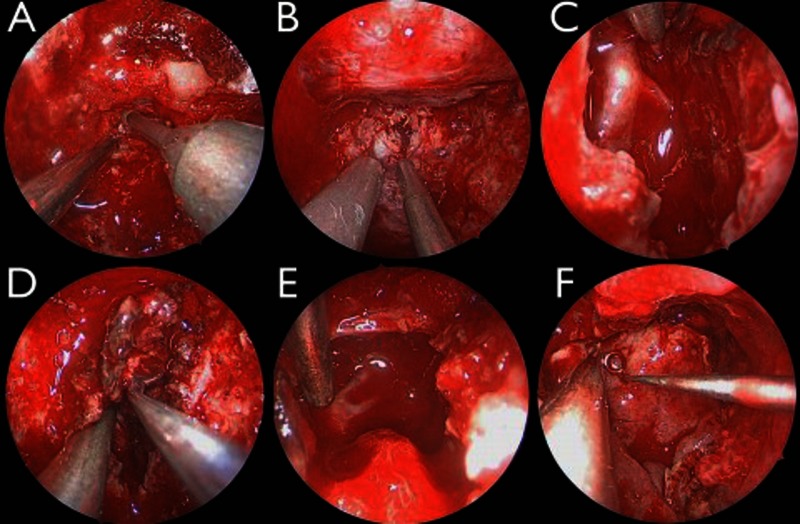
Intraoperative Imaging Via Endoscope A: Transclival approach was started by removing the bone of the upper clivus just under the floor of the sella turcica. B: After the clivectomy was completed, the dura was opened in the midline. C: Finding the basilar and left anterior inferior cerebellar arteries early in the resection. D: Most of the removal of the bulk of the tumor was done with ring curettes. E: Finding the left superior cerebellar and posterior cerebral arteries near in the end of the resection. F: Multi-layered closure with a pedicled nasoseptal flap.

The surgical techniques used for this procedure are demonstrated step-by-step in still frames (Figure 2)

The operation started with harvesting an extended nasoseptal flap (NSF) incorporating the septum, nasal floor, and small portion of the lateral nasal wall. A large ipsilateral maxillary antrostomy was created, and the NSF was displaced into the maxillary sinus to protect until later reconstruction. A posterior septectomy was performed for access, and the anterior face of bilateral sphenoid sinuses was removed, including the rostrum. All intersinus septations were drilled flush, and all sphenoid sinus mucosa was removed. The floor of the sphenoid sinus was drilled flush with the clivus, and a limited upper nasopharyngectomy was performed until adequate access to reach the lower limit of the tumor was obtained as confirmed with stereotactic navigation. Bone overlying the sella was thinned and dissected free.

The clivectomy was started just inferior to the floor of the sella turcica. Bone was removed between the vertical segments of the carotid arteries on each side, and from the dorsum sellae down to the level of the internal acoustic canals. The location of the carotid arteries was determined based on anatomic landmarks and intraoperative navigation. On the left, the pituitary was mobilized extradurally in a superior direction, to facilitate a left posterior clinoidectomy in a manner previous described [5]. The dura was then opened in the midline, and at the superior and inferior margin of the bony opening, it was incised laterally on each side. The tumor was debulked centrally, but as soon as there was enough working space, the inferior pole of the tumor was dissected away from the basilar artery so that it could be protected. The tumor resection subsequently followed an inferior-to-superior path, using the basilar artery as a guide, and was accomplished with tumor aspirator and ring curettes. The left anterior inferior cerebellar artery, superior cerebellar artery, and posterior cerebral artery were freed from the tumor in a sequential fashion. The 45^o^ endoscope was then utilized to guide resection more towards the left, as well as to visualize the superior pole of the tumor. When the reach of the curved ring curettes was at their limit and visualization deteriorated towards the margin of the tumor, resection was halted.

Hemostasis was achieved by patiently repeating injections of Surgiflo^®^ (Johnson & Johnson, New Brunswick, NJ, USA) and cold saline irrigation. Dural closure was performed on multiple levels, first by placing DuraGen^®^ (Integra, Plainsboro, NJ, USA) under the dura in an “inlay” fashion, then by placing a similar piece of DuraMatrix^® ^(Stryker, Kalamazoo, MI, USA) in an "onlay" fashion to cover the entire area of the clivectomy. A piece of abdominal fat was placed on the DuraMatrix^®,^ and the entire skull base defect was covered with the nasoseptal flap harvested at the beginning of the procedure. The operative area was then covered with Surgicel^®^ (Johnson & Johnson), DuraSeal^®^ (Integra), and further bolstered with nasal tampon sponge packing. These surgical maneuvers are shown in Video 1.

**Video 1:** Intraoperative Video This video depicts the step-by-step surgical maneuvers during the surgery

**Figure 3:**
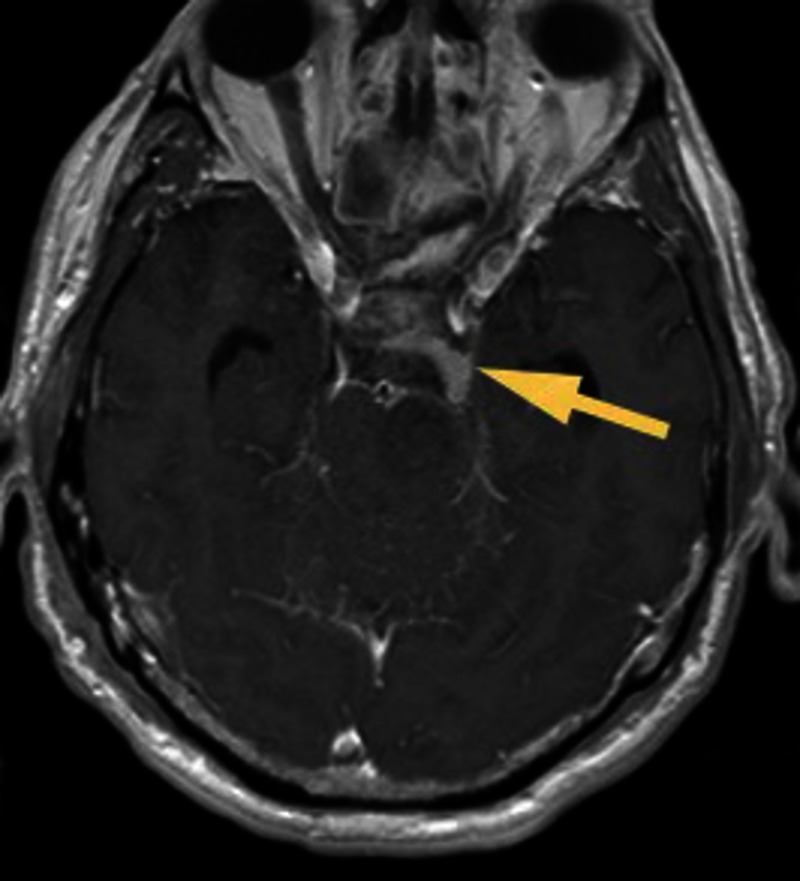
Postoperative MR Imaging Postoperative axial MRI with contrast revealing the degree of resection. The pons is completely decompressed. The arrow is depicting a small tumor residual posterior to the cavernous sinus region.

Postoperatively, the patient experienced right hemiparesis, which resolved over several days. An MRI showed that the majority of the tumor had been removed but there remained a small residual posterior to the left cavernous sinus (Figure 3).

The patient was discharged to rehabilitation on postop day 7 but returned several days later with cerebrospinal fluid rhinorrhea. A reexploration showed that the leak stemmed from the right superior corner of the nasoseptal flap. A piece of abdominal fat was placed here under the nasoseptal flap for better coverage and after three days of lumbar drainage, the patient had no further leakage.

## Discussion

The indications for the endoscopic endonasal approach have continued to expand, ever since the technique was first used for pituitary surgery. The transclival application of the technique to remove midline tumors of the region seemed to be a logical extension of the approach, and indeed, many have reported using this approach for clival chordomas [3, 6]. In contrast, therefore, it is somewhat surprising that there are only 16 reported cases of petroclival meningioma resected with the endoscopic transclival approach [4-5, 7-10]. The reason behind this is probably because, compared to chordomas, meningiomas are more vascular, are frequently situated more laterally in the petroclival region, and thus, increased the difficulty for the midline endonasal endoscopic approach.

Lateral approaches, such as the anterior and posterior petrosectomies, have been considered the preferred ways by many skull base experts to resect petroclival meningiomas [1]. The main disadvantage of these approaches is that the basilar artery, frequently engulfed by the tumor, is seen late in the operation at the distal extreme of the operative field. Protecting this major artery is, therefore, difficult. This weakness of the lateral approaches correlates exactly to the strength of the endoscopic endonasal approach. As seen in our report, the basilar artery was found early in the resection process. Not only was it protected for the majority of the operation, the basilar artery also served as an anatomical guidepost to direct the resection from an inferior-to-superior direction. The endoscopic transclival approach also provides the most direct trajectory to these medially-situated tumors, eliminating brain retraction and minimizing the risk of inadvertent injury to the brainstem.

The transclival approach does provide challenges of its own, as encountered in our experience. Compared to open techniques, it is more difficult to control the bleeding from the meningioma since there is less working space to cauterize the tumor capsule. Preparation is critical to overcoming this hurdle, and the need for intraoperative blood transfusion must be anticipated before the operation starts. Moreover, tumor resection must proceed in an expeditious, but not hasty, manner since the bleeding ends when the tumor bulk is removed. For petroclival meningiomas that extend superior up to the dorsum sellae, such as in our patient, visualization of the superior pole of the tumor might require the upward mobilization of the pituitary gland and a posterior clinoidectomy. Although a transcavernous technique has been designed to accomplish this with minimal risk to pituitary function, this maneuver is technically challenging and further adds to the blood loss [5].

Cerebrospinal fluid leak is always a concern from any endoscopic endonasal approach. Our patient experienced a delayed CSF leak, which is unusual given the multi-layered closure with a vascularized nasoseptal flap that was used for the operation [11]. Fortunately, a minor revision of the closure with an added piece of abdominal fat, coupled with three days of lumbar drainage, solved the leak problem.

**Figure 4:**
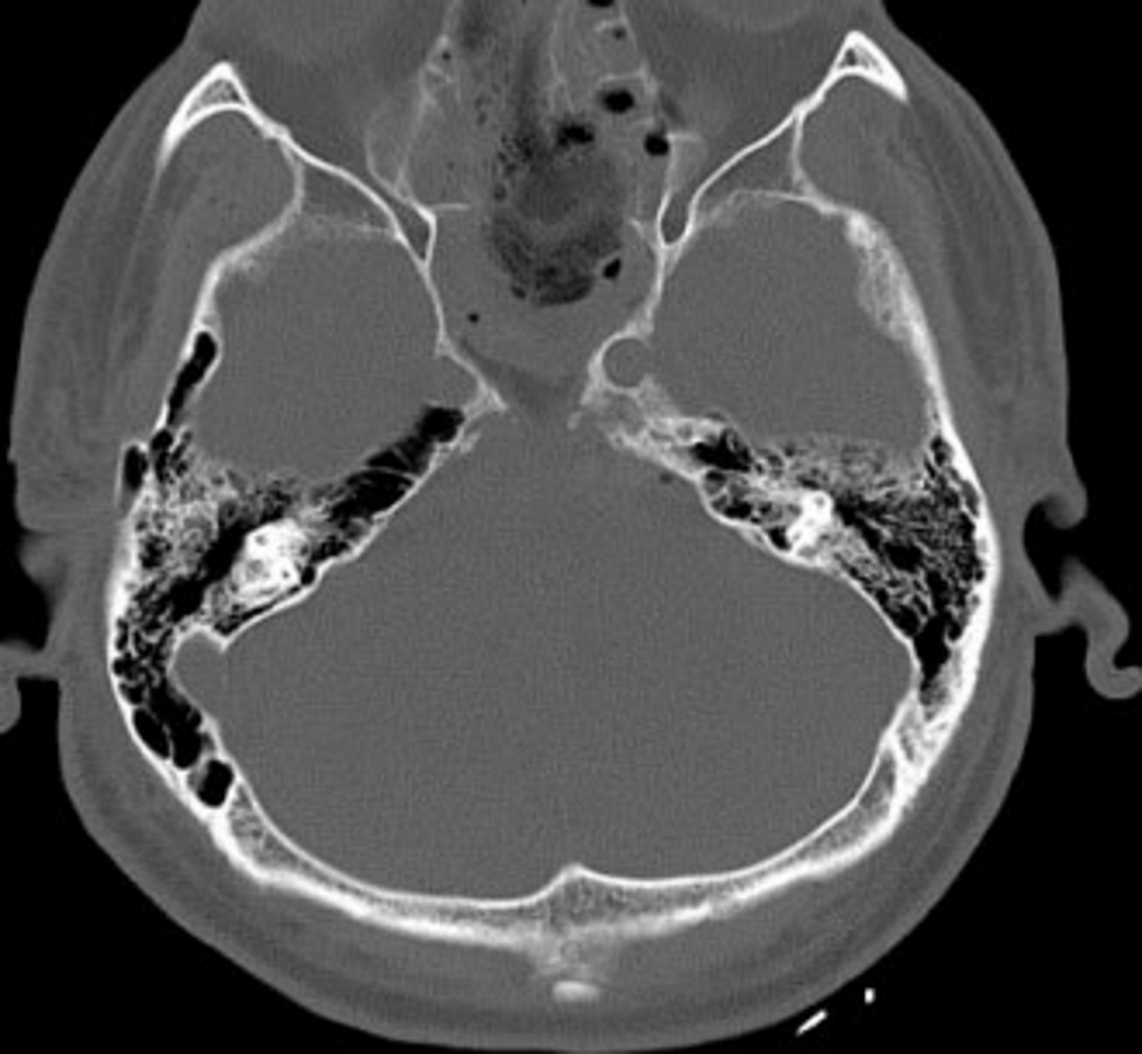
Postoperative CT Imaging Postoperative axial CT showing the extent of the bony clivus resection.

The main disadvantage of a midline approach is the limited extent of the lateral reach. Several recent cadaveric studies have confirmed what seems intuitively apparent, that the endoscopic endonasal transclival approach is inferior to anterior petrosectomy for accessing the lateral petrous ridge [12-13]. As the postoperative MRI of our patient showed, there was a small residual of the tumor out of reach for our approach on the left margin (Figure 3). Two important points can be learned from this. First, patient selection is critical as the transclival approach is not suitable for every petroclival meningioma. If the majority of the meningioma is situated on the petrous bone rather than the clivus, then lateral approaches are still preferable. Secondly, with the refinement of surgical technique and improvement of technology, such as better drills and angled-endoscopes, it will eventually become safer for the patient to add a partial petrosectomy to the transclival approach. As the postoperative CT of our patient showed, additional petrous bone removal posterior to the left carotid would have improved access to the lateral edge of the meningioma and potentially would have allowed a complete resection (Figure 4).

## Conclusions

The endoscopic endonasal transclival approach is ideal for removing medially-situated petroclival meningiomas because of its direct trajectory and avoidance of brain retraction. However, preoperative preparations are key in limiting intraoperative blood loss. In addition, patient selection is also critical since the anatomy of the tumor significantly impacts the likelihood of success. For large tumors with a significant component lateral to the intracavernous carotid artery, the endoscopic approach may not be the best stand-alone option but can be combined with lateral approaches in staged surgical procedures.

## References

[1] Abdel Aziz KM, Sanan A, van Loveren HR, Tew JM Jr, Keller JT, Pensak ML (2000). Petroclival meningiomas: predictive parameters for transpetrosal approaches. Neurosurgery.

[2] Kassam A, Snyderman CH, Mintz A, Gardner P, Carrau RL (2005). Expanded endonasal approach: the rostrocaudal axis. Part II. Posterior clinoids to the foramen magnum. Neurosurg Focus.

[3] Fraser JF, Nyquist GG, Moore N, Anand VK, Schwartz TH (2010). Endscopic endonasal transclival resection of chordomas: operative technique, clinical outcome and review of the literature. J Neurosurg.

[4] Beer-Furlan A, Vellutini EA, Balsalobre L, Stamm AC (2015). Endoscopic endonasal approach to ventral posterior fossa meningioma: From case selection to surgical management. Neurosurg Clin N Am.

[5] Fernandez-Miranda JC, Gardner PA, Rastelli MM Jr, Peris-Celda M, Koutourousiou M, Peace D, Snyderman CH, Rhoton AL Jr (2014). Endoscopic endonasal transcavernous posterior clinoidectomy with interdural pituitary transposition. J Neurosurg.

[6] Stippler M, Gardner PA, Snyderman CH, Carrau RL, Prevedello DM, Kassam AB (2009). Endoscopic endonasal approach for clival chordomas. Neurosurgery.

[7] Alexander H, Robinson S, Wickremesekera A, Wormald PJ (2010). Endoscopic transsphenoidal resection of a mid-clival meningioma. J Clin Neurosci.

[8] Fraser JF, Nyquist GG, Moore N, Anand VK, Schwartz TH (2010). Endoscopic endonasal minimal access approach to the clivus: case series and technical nuances. Neurosurgery.

[9] Prosser JD, Vender JR, Alleyne CH, Solares CA (2012). Expanded endoscopic endonasal approaches to skull base meningiomas. J Neurol Surg B Skull Base.

[10] Khan OH, Anand VK, Schwartz TH (2014). Endoscopic endonasal resection of skull base meningiomas: The significance of a "cortical cuff" and brain edema compared with careful case selection and surgical experience in predicting morbidity and extent of resection. Neurosurg Focus.

[11] Kassam AB, Thomas A, Carrau RL, Snyderman CH, Vescan A, Prevedello D, Mintz A, Gardner P (2008). Endoscopic reconstruction of the cranial base using a pedicled nasoseptal flap. Neurosurgery.

[12] Van Gompel JJ, Alikhani P, Tabor MH, van Loveren HR, Agazzi S, Froelich S, Youssef AS (2014). Anterior inferior petrosectomy: defining the role of endonasal endoscopic techniques for petrous apex approaches. J Neurosurg.

[13] Jacquesson T, Berhouma M, Tringali S, Simon E, Jouanneau E (2015). Which route for petroclival tumors? A comparison between the anterior expanded endoscopic endonasal approach and lateral or posterior routes. World Neurosurg.

